# Risk factors for return to work in colorectal cancer survivors

**DOI:** 10.1002/cam4.3952

**Published:** 2021-05-14

**Authors:** Chung‐Mao Yuan, Chung‐Ching Wang, Wei‐Te Wu, Ching‐Liang Ho, Wei‐Liang Chen

**Affiliations:** ^1^ Department of Internal Medicine Kaohsiung Armed Forces General Hospital Kaohsiung Taiwan; ^2^ Department of Internal Medicine Tri‐Service General Hospital and School of Medicine National Defense Medical Center Taipei Taiwan; ^3^ Division of Environmental Health and Occupational Medicine Department of Family and Community Medicine Tri‐Service General Hospital National Defense Medical Center Taipei Taiwan; ^4^ Division of Family Medicine Department of Family and Community Medicine Tri‐Service General Hospital and School of Medicine National Defense Medical Center Taipei Taiwan; ^5^ National Institute of Environmental Health Sciences National Health Research Institutes Miaoli Taiwan; ^6^ Department of Biochemistry National Defense Medical Center Taipei Taiwan

**Keywords:** colorectal cancer, prognostic factor, retrospective cohort study, return to work

## Abstract

**Background:** The increasing incidence of colorectal cancer among individuals in the productive age‐group has adversely affected the labor force and increased healthcare expenses in recent years. Return to work (RTW) is an important issue for these patients. In this study, we explored the factors that influence RTW and investigated the influence of RTW on survival outcomes of patients with colorectal cancer.

**Methods:** Data of individuals (*N* = 4408) in active employment who were diagnosed with colorectal cancer between 2004 and 2010 were derived from 2 nationwide databases. Subjects were categorized into 2 groups according to their employment status at 5‐year follow‐up. Logistic regression analysis was performed to identify the factors associated with RTW. Survivors were further followed up for another 8 years. Propensity score matching was applied to ensure comparability between the two groups, and survival analysis was performed using the Kaplan–Meier method.

**Results:** In multivariable regression analysis for 5‐year RTW with different characteristics, older age (OR: 0.57 [95% CI, 0.48–0.69]; p < 0.001), treatment with radiotherapy (OR: 0.69 [95% CI, 0.57–0.83]; p < 0.001), higher income (OR: 0.39 [95% CI, 0.32–0.47]; p < 0.001), medium company size (OR: 0.78 [95% CI, 0.63–0.97]; p = 0.022), and advanced pathological staging (stage I, OR: 16.20 [95% CI, 12.48–21.03]; stage II, OR: 13.12 [95% CI, 10.43–16.50]; stage III, OR: 7.68 [95% CI, 6.17–9.56]; p < 0.001 for all) revealed negative correlations with RTW. In Cox proportional hazard regression for RTW and all‐cause mortality, HR was 1.11 (95% CI, 0.80–1.54; p = 0.543) in fully adjusted model.

**Conclusion:** Older age, treatment with radiotherapy, higher income, medium company size, and advanced pathological stage showed negative correlations with RTW. However, we observed no significant association between employment and all‐cause mortality. Further studies should include participants from different countries, ethnic groups, and patients with other cancers.

## Graphical abstract

Older age, treatment with radiotherapy, higher income and advanced pathological stage showed a negative correlation with return to work. No significant association between employment and all‐cause mortality was observed.

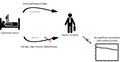

## 1 INTRODUCTION

Progressive population growth and aging have led to increased incidence of cancer and cancer‐associated mortality in recent years.^1^, ^2^ Improved cancer screening and developments in therapeutic modalities have advanced the overall survival rate of cancer patients. This has also contributed to increased diagnosis of cancer in younger age‐groups and an increasing number of cancer survivors in the productive age‐group.^2^, ^3^, ^4^, ^5^ The reduced working ability has an adverse effect on these patients as well as the society at large. Thus, there is an increasing interest in maintaining the employment of cancer survivors.^6^

Colorectal cancer (CRC) is the third most common cancer in the world, accounting for 10.2% of all malignancies; an estimated 1.8 million cases of CRC are newly diagnosed every year.^1^, ^7^ The epidemiological patterns of CRC tend to vary in different parts of the world; however, some distinct trends are observed globally, that is, increases incidence and mortality, decreased mortality rate, and increasing younger age at diagnosis.^1^, ^7^, ^8^, ^9^

Studies have shown that more than half of all cancer survivors avail a period of sick leave for receiving cancer therapy and to cope with the associated disability; in addition, most of these patients returned to work after treatment.^2^, ^5^, ^10^ However, cancer patients were still found to have a higher risk of job loss, less probability of re‐employment, and longer time for returning to work.^3^, ^11^, ^12^ Furthermore, unemployment among cancer survivors was shown to adversely affect their quality of life (QoL); in addition, the reduced household income, declined physical ability and their psychosocial repercussions were shown to influence the prognosis of underlying diseases.^6^, ^13^, ^14^, ^15^ Studies have also shown that being employed inculcates a sense of accomplishment, self‐esteem, and normalcy.^16^, ^17^, ^18^, ^19^ From a societal perspective, the financial implication of resources spent on medical care, welfare, and reduction of the labor force due to absenteeism imposes an extra burden on the government. Therefore, there is increasing awareness of the importance of rehabilitation interventions for cancer survivors to facilitate their return to the work force.^13^, ^20^ However, to the best of our knowledge, no study has directly investigated the correlation between return to work (RTW) and survival outcomes.

Since maintaining the employment is a key concern for cancer patients, identification of factors that influence employment status is imperative. Several studies have explored the factors that influence the employment status among cancer survivors.^13^, ^21^, ^22^, ^23^ Some of these studies have yielded inconsistent results depending on the cancer site or study area. Most studies that have investigated the correlates of change in employment status were based on European and American data. There is a paucity of studies conducted in Asia, which is home to 60% of the global population and accounts for approximately half of all cancer cases and cancer deaths.^1^ In this study, we analyzed the data of employees who were diagnosed with CRC in Taiwan. The aim was to identify factors associated with RTW and to investigate the correlation between RTW and survival outcomes in CRC patients.

## 2 METHODS

### 2.1 Study design

This was a nationwide, retrospective cohort study. Data for this study were derived from two nationwide databases in Taiwan: National Health Insurance Research Database (NHIRD) and Labor Insurance Database (LID). Employees who were diagnosed with CRC between 2004 and 2010 were enrolled initially. Participants were followed up for 5 years after diagnosis of CRC. We analyzed the relationship of various variables with RTW in the 5th year after CRC diagnosis. Subsequently, the surviving patients were divided into RTW and non‐RTW groups depending on their employment status and followed up for another 8 years. Lastly, we compared the survival outcomes in the two groups.

### 2.2 Database

NHIRD is a nationwide database that contains socio‐demographic (e.g., sex, age, residence) and health service‐related information (e.g., health facility, clinical diagnosis, treatment details) of approximately 23 million residents in Taiwan. These data were obtained from National Health Insurance (NHI), an insurance system launched by the Taiwan government in 1995. The NHI had enrolled over 99% of Taiwan's population. In this study, we obtained health‐related information from the NHIRD. Comorbidities and cancer diagnosis were derived according to the International Classification of Diseases, Ninth Revision, Clinical Modification (ICD‐9‐CM) codes.

LID is another nationwide database, which was derived from the labor insurance system in Taiwan. The Taiwan government regulations require mandatory enrolment of all full‐time employees in labor insurance unless they quit their job. This database provides socio‐demographic and labor‐related (e.g., industry, company size, income) information. The industrial classification in LID is according to the industry distribution system, 9th revision of Executive Yuan, Taiwan, which is based on the International Standard Industrial Classification of All Economic Activities (ISIC), revision 4.

### 2.3 Participants

From the NHIRD, we extracted data pertaining to all people aged ≥20 years who were newly diagnosed with CRC between 2004 and 2010. The dataset of CRC was identified according to the International Classification of Diseases for Oncology, third edition (ICD‐O‐3, code C18‐C21). Among these patients, those with other primary malignancies were excluded. Subsequently, we linked the above dataset with LID and selected those individuals whose employment status was “under employment” or “self‐employed” at the time of CRC diagnosis. A total of 4408 full‐time employees were eligible for inclusion.

### 2.4 Outcome measures

The primary outcome of this study was RTW 5 years after CRC diagnosis. Employment status was recorded and checked according to the data in LID. Each participant was followed up until death or the completion of a 5‐year follow‐up. These participants were divided into two groups, “RTW” and “non‐RTW,” based on the employment status at the 5th year after CRC diagnosis. RTW group included the participants who remained in the workforce with or without sick leave after a cancer diagnosis. Individuals who ceased working and did not RTW were classified as a non‐RTW group. The correlates of RTW were analyzed in order to investigate the determinants of RTW in CRC patients.

**FIGURE 1:**
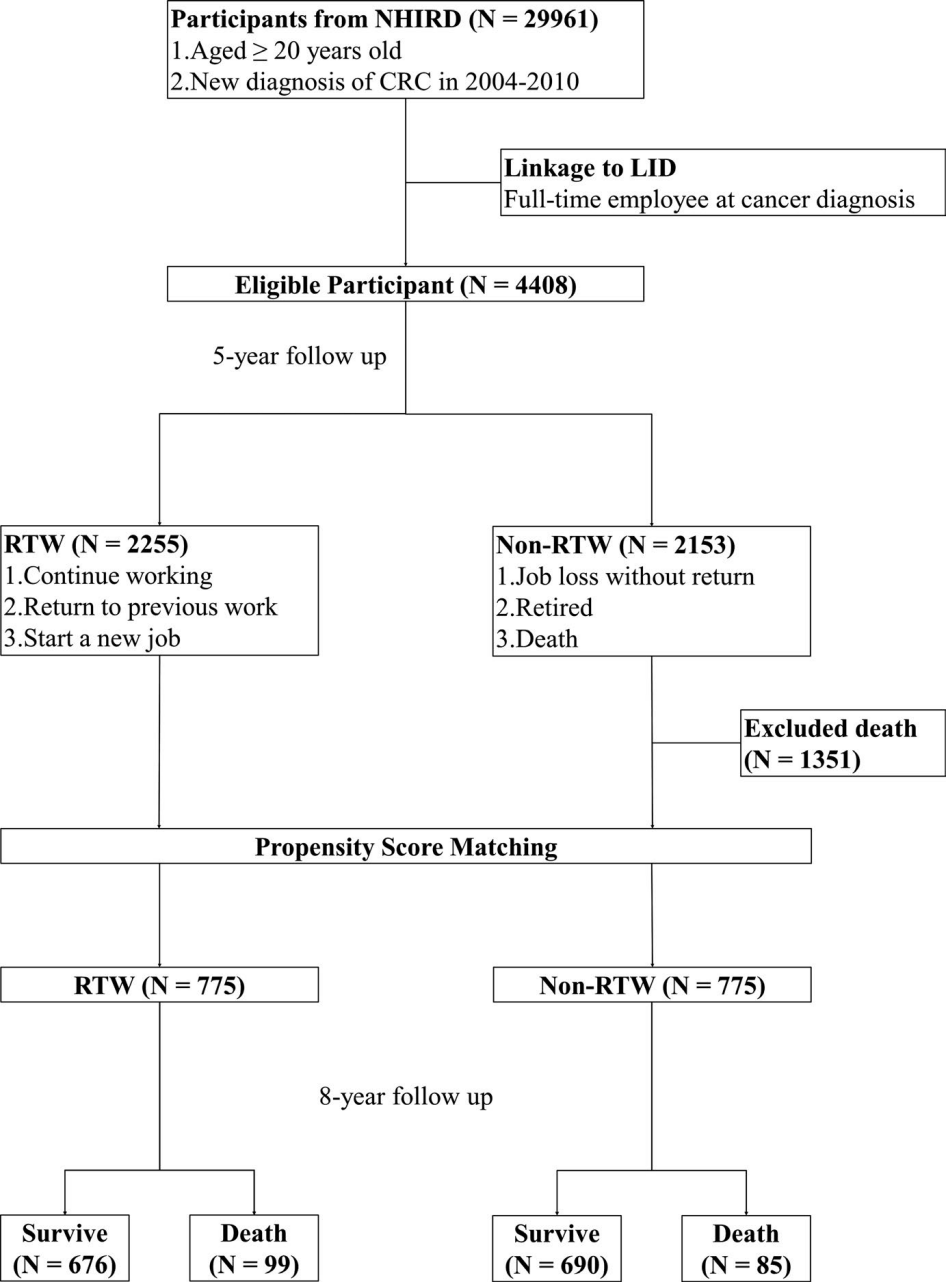
Flowchart of the study protocol

The secondary outcome was long‐term survival. Survival data were acquired through detecting the registration of participants in NHIRD. The surviving participants in the RTW and non‐RTW groups at the 5th year were followed up for another 8 years. We applied propensity score matching in a 1:1 ratio before survival analysis. All‐cause mortality was compared between the RTW and non‐RTW groups to assess the correlation between RTW and survival. The study protocol is shown in Figure 1.

### 2.5 Statistical analysis

The SAS 9.3 (SAS Institute) statistical package was used for data analysis. Continuous and categorical variables are presented as mean ± standard deviation and frequency (percentage), respectively. Between‐group difference with respect to demographic characteristics and comorbid medical disorders were assessed using the independent sample *t*‐test and Chi‐squared test. Univariate and multivariate logistic regression analyses were performed to assess the effect of each demographic characteristic on RTW. Variables that showed a significant association in the univariable model were included in the multivariate model.

In the analysis of all‐cause mortality and RTW, propensity score matching was applied at baseline. Survival analysis was performed using the Kaplan–Meier method and differences between the RTW and non‐RTW groups were assessed using the log‐rank test. Univariate and multivariate Cox proportional hazard regressions were applied. Two‐sided *p* values less than 0.05 were considered indicative of statistical significance.

## 3 RESULTS

### 3.1 Characteristics of the study population

The study population comprised of 4408 employees who were diagnosed with CRC and underwent a 5‐year follow‐up of their employment status. The demographic characteristics of the study population are summarized in Table 1. A total of 2255 participants remained in the work force (1943 worked at the same company and 312 changed their jobs) while 2153 had quit their jobs without return to employment (802 unemployed and 1351 died) in the 5th year after diagnosis of CRC.

**TABLE 1:** Demographic characteristics of study participants

Characteristic	Number of patient (*N* = 4408)	
	*n*	%
Age (years) ± SD (range)	52.8 ± 9.3 (22–86)	
≤45	825	18.7
45–52	1172	26.6
>52	2411	54.7
Gender		
Male	2405	54.6
Female	2003	45.4
Employment status		
Work in same jobs	1943	44.1
Start with new jobs	312	7.1
Jobless	802	18.2
Death	1351	30.6
Comorbidities		
Disorders of lipoid metabolism	445	10.1
Obesity	11	0.2
Alcohol abuse	14	0.3
Hypertension	897	20.3
Myocardial infarction	20	0.5
Congestive heart failure	66	1.5
Peripheral vascular disease	34	0.8
Cerebrovascular disease	99	2.2
Chronic pulmonary disease	167	3.8
Rheumatologic disease	33	0.7
Peptic ulcer disease	617	14
Hemiplegia or paraplegia	14	0.3
Renal disease	67	1.5
Psychoses	19	0.4
Depression	83	1.9
Treatment		
Operation	4277	97
Radiation therapy	665	15.1
Chemotherapy	2031	46.1
Living area		
North	2090	47.4
Central	824	18.7
South	1420	32.2
East	61	1.4
Offshore islands	13	0.3
Income (US dollars)		
≤930	2444	55.4
930–1230	743	16.9
>1230	1221	27.7
Industrial classification		
Agriculture, forestry, fishing, animal, husbandry mining and quarrying	305	6.9
Manufacturing	1367	31
Electricity and gas supply	26	0.6
Water supply and remediation	39	0.9
Construction	505	11.5
Wholesale and retail trade	578	13.1
Transportation and storage	308	7.0
Accommodation and food service	194	4.4
Information and communication	53	1.2
Financial and insurance activities	132	3.0
Real estate activities	44	1.0
Professional, scientific and technology	96	2.2
Support service activities	108	2.5
Public administration and defense	66	1.5
Education	79	1.8
Human health and social work	109	2.5
Amusement and recreation activities	47	1.1
Other service activities	352	8.0
Company size^a^		
Shut down	455	10.3
Small	337	7.6
Medium	986	22.4
Large	2630	59.7
Stage		
I	769	17.4
II	1270	28.8
III	1461	33.1
IV	908	20.6

Abbreviation: SD, standard deviation.

^a^ Company size: small (less than 5 people), medium (less than 200 people in manufacturing, construction, mining, and quarrying; or less than 100 people in other industries), large (more than 200 people in manufacturing, construction, mining, and quarrying; or more than 100 people in other industries).

### 3.2 Associations between RTW and different characteristics

Table 2 shows the univariable odds ratios (ORs) for 5‐year RTW associated with different characteristics. RTW showed a negative correlation with older age (OR: 0.73 [95% CI, 0.62–0.85]; *p* < 0.001), male sex (OR: 0.76 [95% CI, 0.67–0.85]; *p* < 0.001), comorbid hypertension (OR: 0.82 [95% CI, 0.71–0.95]; *p* = 0.007) and cerebrovascular disease (OR: 0.41 [95% CI, 0.26–0.63]; *p* < 0.001), treatment with radiotherapy (OR: 0.79 [95% CI, 0.67–0.93]; *p* = 0.004) and chemotherapy (OR: 0.62 [95% CI, 0.55–0.69]; *p* < 0.001), higher income (OR: 0.47 [95% CI, 0.41–0.54]; *p* < 0.001), occupation electricity and gas supply (OR: 0.35 [95% CI, 0.13–0.99]; *p* = 0.049), and shut down (OR: 0.77 [95% CI, 0.63–0.94]; *p* = 0.009) and medium (OR: 0.86 [95% CI, 0.74–0.99]; *p* = 0.037) company size. Conversely, treatment with operation (OR: 1.56 [95% CI, 1.10–2.23]; *p* = 0.014), living in central Taiwan (OR: 1.23 [95% CI, 1.03–1.46]; *p* = 0.019), and lower pathological stage (stage I, OR: 12.80 [95% CI, 10.07–16.25]; stage II, OR: 10.86 [95% CI, 8.73–13.49]; stage III, OR: 6.58 [95% CI, 5.33–8.13]; *p* < 0.001 for all) demonstrated a positive association with RTW.

**TABLE 2:** Univariate logistic regression for RTW by 5 year

Characteristic	OR	95% CI	*p* value
Age (years)			
≤45^a^			
45–52	0.99	(0.83, 1.19)	0.981
>52	0.73	(0.62, 0.85)	<.001***
Gender			
Male	0.76	(0.67, 0.85)	<.001***
Female^a^			
Comorbidities			
Disorders of lipoid metabolism	0.98	(0.81, 1.20)	0.869
Obesity	0.36	(0.10, 1.35)	0.129
Alcohol abuse	0.38	(0.12, 1.22)	0.103
Hypertension	0.82	(0.71, 0.95)	0.007**
Myocardial infarction	0.78	(0.32, 1.89)	0.582
Congestive heart failure	0.66	(0.40, 1.08)	0.096
Peripheral vascular disease	1.08	(0.55, 2.11)	0.835
Cerebrovascular disease	0.41	(0.26, 0.63)	<.001***
Chronic pulmonary disease	0.94	(0.69, 1.28)	0.701
Rheumatologic disease	1.02	(0.51, 2.01)	0.967
Peptic ulcer disease	0.87	(0.73, 1.03)	0.106
Hemiplegia or paraplegia	0.38	(0.12, 1.22)	0.103
Renal disease	0.68	(0.42, 1.11)	0.124
Psychoses	1.06	(0.43, 2.62)	0.898
Depression	1.03	(0.67, 1.59)	0.905
Treatment			
Operation	1.56	(1.10, 2.23)	0.014*
Radiation therapy	0.79	(0.67, 0.93)	0.004**
Chemotherapy	0.62	(0.55, 0.69)	<.001***
Living area			
North	0.98	(0.86, 1.13)	0.804
Central	1.23	(1.03, 1.46)	0.019*
South^a^			
East + offshore islands	1.33	(0.83, 2.12)	0.235
Income (US dollars)			
≤930^a^			
930–1230	1.08	(0.92, 1.28)	0.361
>1230	0.47	(0.41, 0.54)	<.001***
Industrial classification			
Agriculture, forestry, fishing, animal, husbandry mining and quarrying	1.14	(0.62, 2.12)	0.667
Manufacturing	1.02	(0.57, 1.82)	0.953
Electricity and gas supply	0.35	(0.13, 0.99)	0.049*
Water supply and remediation	0.48	(0.20, 1.15)	0.100
Construction	0.95	(0.52, 1.72)	0.858
Wholesale and retail trade	1.03	(0.57, 1.87)	0.912
Transportation and storage	0.78	(0.43, 1.48)	0.473
Accommodation and food service	1.13	(0.60, 2.14)	0.706
Information and communication	1.07	(0.49, 2.36)	0.860
Financial and insurance activities	0.99	(0.51, 1.92)	0.971
Real estate activities	1.15	(0.50, 2.62)	0.740
Professional, scientific and technology	0.96	(0.48, 1.93)	0.905
Support service activities	1.03	(0.52, 2.05)	0.928
Public administration and defense	0.96	(0.45, 2.06)	0.911
Education	1.21	(0.58, 2.49)	0.614
Human health and social work	0.98	(0.49, 1.94)	0.945
Amusement and recreation activities^a^			
Other service activities	1.12	(0.61, 2.07)	0.701
Company size^b^			
Shut down	0.77	(0.63, 0.94)	0.009**
Small	1.07	(0.85, 1.35)	0.554
Medium	0.86	(0.74, 0.99)	0.037*
Large^a^			
Stage			
I	12.80	(10.07, 16.25)	<.001***
II	10.86	(8.73, 13.49)	<.001***
III	6.58	(5.33, 8.13)	<.001***
IV^a^			

Abbreviations: CI, confidence interval; OR, odds ratio; RTW, return to work.

^a^ Reference category.

^b^ Company size: small (less than 5 people), medium (less than 200 people in manufacturing, construction, mining, and quarrying; or less than 100 people in other industries), large (more than 200 people in manufacturing, construction, mining, and quarrying; or more than 100 people in other industries).

* *p* <  0.05 for comparison between RTW and non‐RTW participants.

** *p* < 0.01 for comparison between RTW and non‐RTW participants.

*** *p* < 0.001 for comparison between RTW and non‐RTW participants.

The statistically significant variables (age, gender, treatment, living area, income, company size, and pathological stage) were included in multivariable regression analysis (Table 3). Age, treatment, living area, income, company size, and pathological stage showed statistically significant difference. Older age (OR: 0.57 [95% CI, 0.48–0.69]; *p* < 0.001), treatment with radiotherapy (OR: 0.69 [95% CI, 0.57–0.83]; *p* < 0.001), higher income (OR: 0.39 [95% CI, 0.32–0.47]; *p* < 0.001), and medium company size (OR: 0.78 [95% CI, 0.63–0.97]; *p* = 0.022) revealed a negative correlation with RTW, whereas living in east and offshore island of Taiwan (OR: 1.85 [95% CI, 1.05–3.25]; *p* < 0.001) and lower pathological staging (stage I, OR: 16.20 [95% CI, 12.48–21.03]; stage II, OR: 13.12 [95% CI, 10.43–16.50]; stage III, OR: 7.68 [95% CI, 6.17–9.56]; *p* < 0.001 for all) indicated a positive correlation with RTW.

**TABLE 3:** Multivariate logistic regression for RTW by 5 years

Characteristic	OR	95% CI	*p* value
Age (years)			
≤45^a^			
45–52	0.93	(0.76, 1.14)	0.488
>52	0.57	(0.48, 0.69)	<.001***
Gender			
Male	0.87	(0.76, 1.01)	0.059
Female^a^			
Treatment			
Operation	1.47	(0.98, 2.19)	0.061
Radiation therapy	0.69	(0.57, 0.83)	<.001***
Chemotherapy	0.92	(0.80, 1.07)	0.277
Living area			
North	0.97	(0.83, 1.13)	0.721
Central	1.18	(0.97, 1.44)	0.095
South^a^			
East + offshore islands	1.85	(1.05, 3.25)	0.032*
Income (US dollars)			
≤930^a^			
930–1230	1.09	(0.90, 1.32)	0.381
>1230	0.39	(0.32, 0.47)	<.001***
Company size^b^			
Shut down	0.78	(0.60, 1.03)	0.084
Small	0.89	(0.65, 1.21)	0.454
Medium	0.78	(0.63, 0.97)	0.022*
Large^a^			
Stage			
I	16.20	(12.48, 21.03)	<.001***
II	13.12	(10.43, 16.50)	<.001***
III	7.68	(6.17, 9.56)	<.001***
IV^a^			

Abbreviations: CI, confidence interval; OR, odds ratio; RTW, return to work.

^a^ Reference category.

^b^ Company size: small (less than 5 people), medium (less than 200 people in manufacturing, construction, mining, and quarrying; or less than 100 people in other industries), large (more than 200 people in manufacturing, construction, mining, and quarrying; or more than 100 people in other industries).

* *p* < 0.05 for comparison between RTW and non‐RTW participants.

*** *p* < 0.001 for comparison between RTW and non‐RTW participants.

### 3.3 Association of RTW with all‐cause mortality

To assess the influence of RTW on survival, we analyzed the correlation between RTW and all‐cause mortality. After propensity score matching, there were 775 participants each in the RTW and non‐RTW groups. Table 4 shows the demographic characteristics of the propensity score‐matched cohort.

**TABLE 4:** Demographic characteristics of RTW and non‐RTW groups after propensity score matching

Characteristic	Total (*N* = 1550)	RTW (*N* = 775)		Non‐RTW (*N* = 775)		*p* value
		*n*	%	*n*	%	
Age (years) ± SD (range)	54.8 ± 8.7 (22–86)	54.1 ± 8.3 (22–82)		55.4 ± 9.0 (27–86)		0.842
≤45	198	99	12.8	99	12.8	
45–52	305	148	19.1	157	20.3	
>52	1047	528	68.1	519	67.0	
Gender						1.000
Male	968	484	62.5	484	62.5	
Female	582	291	37.5	291	37.5	
Comorbidities						
Disorders of lipoid metabolism	180	89	11.5	91	11.7	0.874
Obesity + hemiplegia or paraplegia	7	3	0.4	4	0.5	1.000
Alcohol abuse	6	3	0.4	3	0.4	1.000
Hypertension	353	171	22.1	182	23.5	0.505
Myocardial infarction	7	3	0.4	4	0.5	1.000
Congestive heart failure	26	12	1.5	14	1.8	0.692
Peripheral vascular disease	21	11	1.4	10	1.3	0.826
Cerebrovascular disease	37	17	2.2	20	2.6	0.618
Chronic pulmonary disease	51	28	3.6	23	3.0	0.477
Rheumatologic disease	9	5	0.6	4	0.5	1.000
Peptic ulcer disease	219	106	13.7	113	14.6	0.610
Renal disease	19	9	1.2	10	1.3	0.817
Psychoses	8	4	0.5	4	0.5	1.000
Depression	24	14	1.8	10	1.3	0.411
Treatment						
Operation						
No	40	18	2.3	22	2.8	0.522
Yes	1510	757	97.7	753	97.1	
Radiation therapy						
No	1322	670	86.5	652	84.1	0.197
Yes	228	105	13.5	123	15.9	
Chemotherapy						
No	878	485	62.6	393	50.7	<.001***
Yes	672	290	37.4	382	49.3	
Living area						0.419
North	796	390	50.3	406	52.4	
Central	242	133	17.2	109	14.1	
South	492	240	31.0	252	32.5	
East + offshore islands	20	12	1.5	8	1.0	
Income (US dollars)						0.757
≤930	556	275	35.5	281	36.3	
930–1230	212	111	14.3	101	13.0	
>1230	782	389	50.2	393	50.7	
Industrial classification						0.377
Agriculture, forestry, fishing, animal, husbandry mining and quarrying	88	48	6.2	40	5.2	
Manufacturing	519	251	32.4	268	34.6	
Electricity and gas supply	18	6	0.8	12	1.5	
Water supply and remediation	16	5	0.6	11	1.4	
Construction	148	71	9.2	77	9.9	
Wholesale and retail trade	212	111	14.3	101	13.0	
Transportation and storage	130	62	8.0	68	8.8	
Accommodation and food service	52	23	3.0	29	3.7	
Information and communication	26	14	1.8	12	1.5	
Financial and insurance activities	62	38	4.9	24	3.1	
Real estate activities	17	8	1.0	9	1.2	
Professional, scientific and technology	36	14	1.8	22	2.8	
Support service activities	24	12	1.5	12	1.5	
Public administration and defense	23	10	1.3	13	1.7	
Education	25	15	1.9	10	1.3	
Human health and social work	42	22	2.8	20	2.6	
Amusement and recreation activities	12	5	0.6	7	0.9	
Other service activities	100	60	7.7	40	5.2	
Company size^a^						0.476
Shut down	171	89	11.5	82	10.6	
Small	127	71	9.1	56	7.2	
Medium	385	191	24.6	194	25	
Large	867	424	54.7	443	57.2	
Stage						0.998
I	363	183	23.6	180	23.2	
II	569	283	36.5	286	36.5	
III	516	258	33.3	258	33.3	
IV	102	51	6.6	51	6.6	

Abbreviations: RTW, return to work; SD, standard deviation.

^a^ Company size: small (less than 5 people), medium (less than 200 people in manufacturing, construction, mining and quarrying; or less than 100 people in other industries), large (more than 200 people in manufacturing, construction, mining and quarrying; or more than 100 people in other industries).

*** *p* < 0.001 for comparison between RTW and non‐RTW participants.

**FIGURE 2:**
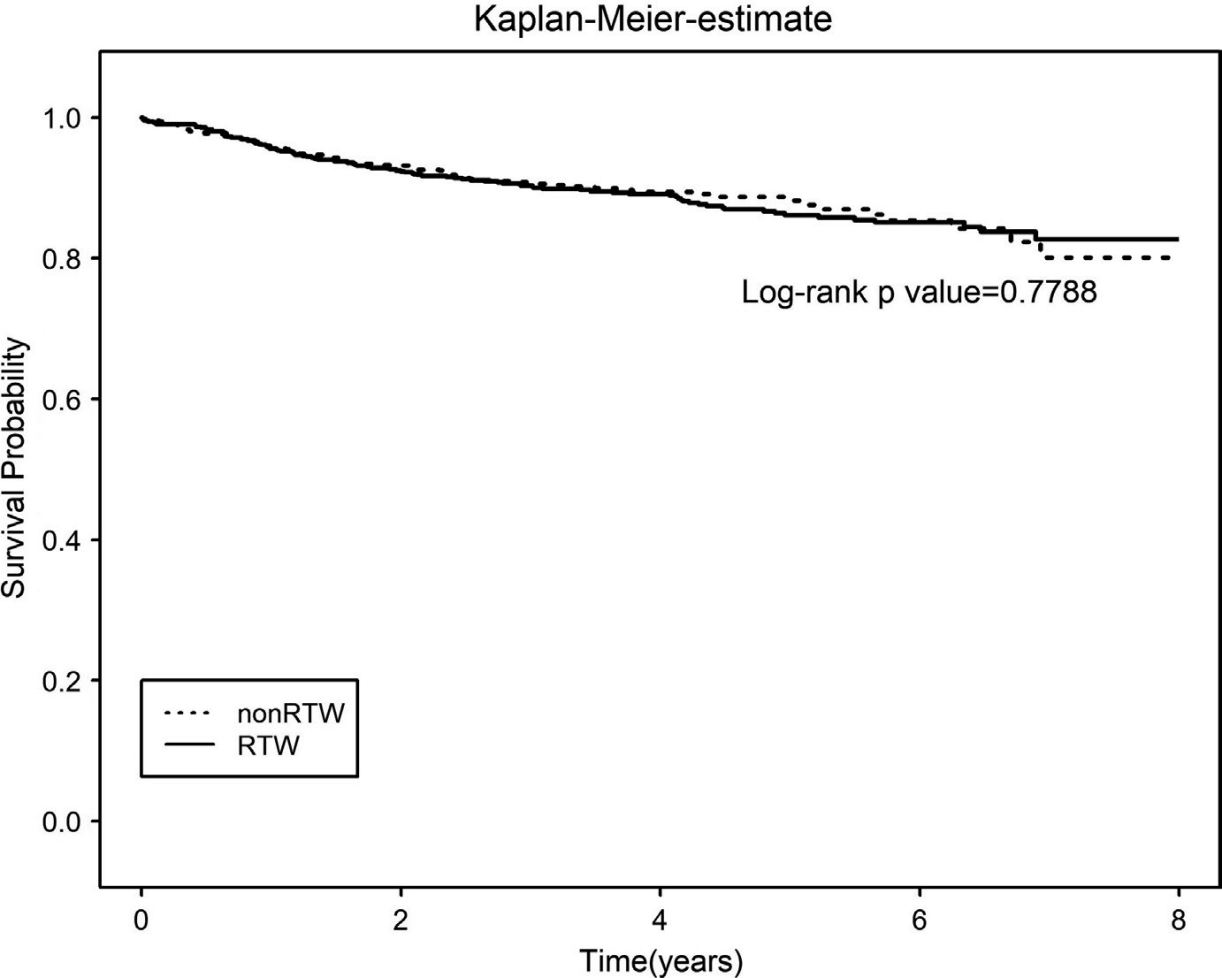
Kaplan–Meier curve for all‐cause mortality

The result of Cox proportional hazard regression for RTW and all‐cause mortality was presented in hazard ratios (HRs). HR was 0.94 (95% CI, 0.70–1.25; *p* = 0.652) in unadjusted model, and 1.11 (95% CI, 0.80–1.54; *p* = 0.543) in fully adjusted model. Figure 2 showed the result of survival analysis in Kaplan‐Meier plot. No statistically significant difference was observed in all‐cause mortalities among RTW and non‐RTW groups.

## 4 DISCUSSION

There were two main objectives of this study. The first objective was to assess the impact of demographic characteristics, health‐related variables, and labor‐related variables on RTW. The second objective was to assess the correlation between RTW and long‐term survival of CRC survivors.

Among the characteristics that influenced employment status, age, gender, comorbidity (hypertension and cerebrovascular disease), treatment, living area, income, occupation, company size, and pathological stage showed a significant difference between RTW and non‐RTW groups by 5 years after CRC diagnosis in the univariate logistic regressions model. This finding was consistent with previous studies that investigated changed in working status among cancer survivors.^13^, ^23^, ^24^, ^25^ However, after adjusting for other variables in multivariate logistic regression, only age, treatment, living area, income, company size, and pathological stage showed a significant correlation with employment status. Many studies have identified factors that influence post‐cancer employment change. In a systemic review by Sze Loon Chow et al. (2014), these factors were categorized into personal, health, financial, and environmental factors.^13^ To integrate these findings, we can identify some common factors that affect the RTW.

First, financial issue was the primary concern that made patients RTW.^6^, ^17^, ^18^, ^26^ Irrespective of the cancer type and demographic characteristics, most cancer survivors indicated financial pressure as their primary consideration while deciding whether to continue and RTW.^18^, ^27^ Apart from income, the role of insurance has also been widely discussed. Adequate health insurance provides financial support, which increases the affordability of medical expenses and allows patients to take time off for their cancer therapy without the apprehension of being unemployed. Furthermore, some studies revealed the correlation between marital status and change in employment status, which was also attributed to financial considerations. Married persons were shown less likely to RTW than singles as that they may have financial support from their partners.^28^ On the contrary, people who were the only or the main source of income in their family are likely to experience greater financial pressure.^26^

Second, RTW is also based on adequate physical condition and working ability. The poorer the physical status, the less is the probability of RTW. Although there were no quantified performance status variables such as Eastern Cooperative Oncology Group (ECOG) or Karnofsky performance score in this study, some previous studies have found that the impact of cancer type, staging, comorbidity, and treatment decision on change in employment may reflect the patients' physical status.^10^, ^13^, ^21^, ^29^ Decline in the ability to perform work and activities of daily living are a barrier for patients seeking a return to employment. Some patients chose to retire from their work after cancer diagnosis, while others RTW after perceiving the adequacy of their physical status.^18^

Third, psychosocial factors also have an important influence on the decision to RTW. These factors include family, workplace environment, and the patients' mental status. We did not investigate these aspects in the present study. An exploratory study investigated the RTW experience of cancer patients, by performing patient interviews. The study elicited several considerations of patients.^18^ Some patients went back to their work to acquire a sense of normality, while others returned to work due to their perceived sense of responsibility and feeling of loyalty toward their work. Studies have also indicated the importance of support from the employers and colleagues.^30^

Table 5 highlights the facilitators and barriers for employment status identified in studies that included CRC patients.^10^, ^12^, ^21^, ^22^, ^24^, ^25^, ^28^, ^31^, ^32^, ^33^, ^34^, ^35^ The present study had a distinctly large sample size (*N* = 4408). Lower income and undergoing surgery were identified as facilitators for employment and RTW, whereas older age, male sex, and advanced pathological stage were identified as barriers to employment and RTW. Income reflected a person's financial ability. Patients with higher income are likely to be more financially secure. In contrast, those with lower income might be forced to RTW as soon as possible due to their financial constraints. Advanced disease represents poorer physical activity, which imposed a burden on cancer survivors returning to their work. The impact of age on RTW is determined by both financial factors and physical ability. In general, aging is associated with the decline in physical condition. Furthermore, elderly tend to have better financial stability than middle‐aged and young people. Both these aspects explain the negative correlations between age and RTW.

**TABLE 5:** Review literature

Study	Year	Country	Study Design	Participants with CRC	Variables	Facilitator for employment & RTW	Barrier of employment & RTW
Our study	2021	Taiwan	Retrospective cohort study	*N* = 4408	RTW	Lower pathological stage	Older age Higher income Radiotherapy Medium company size
Den Bakker CM et al.	2020	Netherlands	Retrospective cohort study	*N* = 317	RTW	(1 year after sick leave) No mentioned	Metastases Emotional distress Postoperative complications Stoma Adjuvant treatment
						(2 years after sick leave) Small company size (<10)	Metastases Emotional distress Postoperative complications
Den Bakker CM et al.	2018	Worldwide	Systemic Review	*N* = 12,800 (8 studies, *N* ranging from 50 to 4343)	RTW	No mentioned	(Neo)adjuvant therapy Higher age Co‐morbidities
					Work disability	No mentioned	Previous unemployment Extensive surgical resection Postoperative complications
LJ Chen et al.	2016	Sweden	Prospective cohort study	*N* = 3438	Unemployment (Work loss)	Anterior resection	Prediagnostic work loss Neoadjuvant therapy Advanced stage Relapse‐free patients Surgical complications Abdominoperineal resection
Mehnert A et al.	2013	Germany	Prospective cohort study	*N* = 42	RTW Time to RTW	Intention to RTW Perceived employer accommodation High job requirement Sick leave absence	Cancer recurrence Cancer metastasis Problematic social interaction Higher UICC cancer stage
Torp S et al.	2013	Norway	Cross‐sectional registry study	*N* = 164	Employment rate	Lower age Higher income Higher education	Sick leave >30 days Cancer (female) Single (male) No children (male)
Yarker J et al.	2010	U.K.	Qualitative study	*N* = 1	Experience of RTW	Communication and support from occupational health, line manager, and colleagues	Delayed impact of cancer Decline ability of work Wear‐off effect of support
Paraponaris A et al.	2010	France	Cross‐sectional survey	*N* = 121	Unemployment (Job tenure)	Higher social‐professional status Higher Education (male) Higher income (male)	Workplace discrimination Fix‐term contrast (female)
Earle CC et al.	2010	U.S.A.	Prospective cohort study	*N* = 1610	Unemployment	Better education	Advanced stage Married women (lower income) Older age (higher income)
Park JH et al.	2009	Korea	Prospective cohort study	1st baseline *N* = 585	Time to job loss	No mentioned	Cancer
				2nd baseline *N* = 160	Time to RTW	No mentioned	Cancer
Gordon L	2008	Australia	Cohort study	*N* = 975	Unemployment (Work cessation)	Private health insurance	Fewer work hours Older age (male) Radiotherapy (male) Chemotherapy (female)
Park JH et al.	2007	Korea	Retrospective cohort study	1st baseline *N* = 585	Time to job loss	No mentioned	Female Younger (<30) or older (>50) Company employees Lower income
				2nd baseline *N* = 160	Time to RTW	No mentioned	Female Older (>50)
Short PF et al.	2005	U.S.A.	Cross‐sectional survey	*N* = 96	Unemployment Disability rate	Postgraduate education Early stage at diagnosis	Women Under initial treatment New cancer or metastasis Advanced stage at diagnosis Chronic health condition

Abbreviation: RTW, return to work.

Of note, the observed influence of “income” on employment status in our study was not consistent with the result of previous studies. In our study, lower income was found to be a facilitator for RTW; however, other studies have yielded opposite findings.^21^, ^32^, ^34^ This discrepancy is likely attributable to economic factors peculiar to Taiwan. Due to NHI coverage, health care and medical treatment in Taiwan is less expensive than that in most other countries. The financial stress in Taiwan is mainly reflected to the reduced productivity due to sick leave or job loss, which increases the need for survivors with lower income to RTW. On the other hand, financial stress in other countries is mainly due to the medical expenses. Patients with higher income are more likely to receive better treatment, which explains the better outcomes and better preserved ability for working. However, there were no standard criteria to define income level in previous studies. Future studies with standardization of income level strata are required to identify correlation between income level and subsequent employment status.

Apart from the factors that affect employment status, very few studies have investigated the influence of RTW on cancer survivors. In this study, we investigated the correlation between RTW and survival of CRC patients in Taiwan. We believe that the better survival of patients who RTW may be attributable to the following reasons. First, work ability is influenced by a combination of individuals' physical, psychological, and social resources.^2^ Patient who RTW are likely to have better physical and mental status, which is liable to contribute to better survival outcomes. Second, RTW may have a positive influence on the physical and mental health of patients. Mahar et al. found that patients who continued working showed better physical and mental functioning, QoL, and lower psychosocial distress than patients who RTW with sick leave and patients who discontinued working after cancer diagnosis.^36^

However, in this study, we observed no significant difference in all‐cause mortality between RTW and non‐RTW groups. This may be attributable to minimization of selection bias after the use of statistical techniques such as propensity score matching. The similar baseline characteristics in both groups may have annulled the influence of better physical and mental status on survival in the RTW group. Nevertheless, we did not evaluate other outcomes such as QoL, physical function, or psychosocial status between the RTW and non‐RTW groups. The impact of RTW on outcomes among cancer survivors remains uncertain.

A key limitation of this study was that we grouped the participants according to their employment status at the time of follow‐up, which means that randomization was unavailable in our study. Other limitations include the lack of quantified performance status data and the absence of tools to evaluate the quality of RTW. Moreover, the outcome measure was confined to survival and we did not measure other indices such as QoL. Lastly, the study population exclusively comprised of CRC patients in Taiwan. Future studies including participants from different countries and ethnic groups, and patients with other cancers are required to elucidate the impact of RTW on cancer survival.

## CONFLICT OF INTEREST

The authors declared that no competing interests exist.

## ETHICS APPROVAL

The study protocol was approved by the Institutional Review Board of Tri‐Service General Hospital, National Defense Medical Center (IRB no. 1‐107‐05‐129) and performed in accordance with the Declaration of Helsinki.

## Data Availability

The data that support the findings of this study are available from Taiwan National Health Insurance Research Database (NHIRD) and Taiwan Labor Insurance Database (LID). Restrictions apply to the availability of these data, which were used under license for this study. Data are available from the authors with the permission of NHIRD and LID.
